# Family Communication Problems, Psychosocial Adjustment and Cyberbullying

**DOI:** 10.3390/ijerph16132417

**Published:** 2019-07-08

**Authors:** Ana Romero-Abrio, Belén Martínez-Ferrer, Daniel Musitu-Ferrer, Celeste León-Moreno, María Elena Villarreal-González, Juan Evaristo Callejas-Jerónimo

**Affiliations:** 1 Department of Education and Social Psychology, Pablo Olavide University, 41013 Seville, Spain; 2 Faculty of Psychology, Autonomous University of Nuevo Leon, Monterrey 64460, Nuevo Leon, Mexico

**Keywords:** cyberbullying, family communication, psychological distress, attitude towards institutional authority, problematic use of social networking sites

## Abstract

The aim of the present study was to analyze the relationship between family communication problems and cyberbullying, through psychosocial adjustment—psychological distress, attitude towards institutional authority, and problematic use of social networking sites—in adolescents. Random sampling by conglomerates was performed. A total of 8115 adolescents participated in the study (51.5% boys, 49.5% girls), and were aged between 11 and 16 years old (M = 13.34, SD = 1.04) and enrolled in the State of Nuevo León (Mexico). A structural equations model was developed using the Structural Equation Modeling Software (EQS). The results showed that problematic family communication is directly associated with cyberbullying, and also indirectly through the relationships of psychological distress and attitude towards transgression of social norms with the problematic use of social networking sites. The multi-group analyses also revealed gender differences in these relationships. Finally, the obtained results were discussed and their practical implications were shown.

## 1. Introduction

Cyberbullying is a growing social problem, particularly among adolescents and with a higher prevalence in developed countries [1,2,3]. The emergence of information and communication technologies (ICTs) has increased bullying behavior among adolescents, especially in the last decade [2,4]. Bullying was defined by Olweus [5] as aggressive and intentional behavior that is carried out by a group or an individual, repeatedly and over time, against a victim who cannot easily defend himself. Furthermore, bullying implies an imbalance of power [6]. Cyberbullying is perpetrated through technological media [7], and differs from bullying in that ICTs allow anonymity and physical distance between the aggressor and the victim, which is associated with a disconnection with the victim’s suffering [8].

The existing concern regarding this problem has prompted an increase in the number of studies analyzing cyberbullying and its relationship with psychosocial variables, taking into account different contexts of adolescent socialization [7,9,10]. Previous studies have highlighted the importance of different aspects of family relationships in the analysis of cyberbullying [11], such as family functioning [12], parental socialization styles [13,14], and family atmosphere [3,15]. One highly relevant variable in this context is child–parent communication, which is a way of transmitting affection and emotional bonding and is also an essential component of family functioning [6,16] and a predictor of adolescents’ psychosocial adjustment [17,18].

It has been observed that open family communication strengthens the child–parent relationship, which serves as a protective factor against cyberbullying [19,20,21], whereas cyber-offenders and cybervictims have the perception that communication with their parents is more problematic [22]. Attention has recently been drawn to the importance of analyzing other factors to explain the link between family communication and cyberbullying [8]. The present study incorporates psychological distress, attitude towards the transgression of social norms, and problematic use of social networking sites (PUSNS).

Adolescence is an evolutionary period especially vulnerable to the development of psychological and mental health problems [23]. One of these problems, psychological distress, is defined as unpleasant feelings and emotions expressed during symptoms of anxiety and depression, and which usually leads to impaired psychological functioning at the behavioral, cognitive and emotional level [16,23,24]. In the family environment, it has been observed that that psychological distress is related to problematic parent–child communication [25,26]. Also, other studies have confirmed that adolescents involved in cyberbullying behaviors present symptoms of psychological distress [27,28], particularly cybervictims [29,30]. This relationship could be attributed to the fact that cybervictims, when faced with situations of harassment, tend to isolate themselves from both their peers and families, resulting implicitly in emotional problems such as anxiety and depression [31,32].

Another variable that is considered to be relevant and that can help explain cyberbullying behaviors is attitude towards institutional authority. Previous studies have reported that adolescents with behavioral problems show more unfavorable attitudes towards figures of authority, such as families and teachers [17,33,34]. In the family sphere, these attitudes are related to the quality of communication between parents and children [16]. Indeed, open communication is associated with a positive attitude towards institutional authority [35,36,37]. It has also been found that adolescents who show a positive attitude towards institutional authority are less involved in situations of peer violence [38,39] and cyberbullying [40].

Finally, since cyberbullying occurs through technological means, the authors consider it worthwhile to explore other variables relating to PUSNS [41,42,43]. PUSNS is defined as an individual being overly concerned about social networking sites (SNSs), to be driven by a strong motivation to log on or use SNSs, and to devote so much time and effort to SNSs that it impairs other social activities, studies or work, interpersonal relationships, and physical and mental health and wellbeing [44,45,46]. This problematic use is related to the expression of violent behavior in different contexts, such as bullying [47], peer violence [46], and child-to-parent violence [33]. Other studies have also highlighted the relationship of PUSNS with cyberbullying [48] and cybervictimization [49,50]. One recent study [51] reported that adolescents with greater PUSNS were more frequently involved in cyberbullying behaviors and that the strongest predictors of PUSNS was depressive symptomatology which, in turn, is one of the most important indicators of psychological distress.

In terms of gender, previous studies have also indicated that gender is a relevant variable when problematic online behaviors and cyberbullying are considered. Thus, girls more often use social networking sites that allow them to communicate with their peers [51,52] whereas boys spend more time on other activities such as video games or visiting web pages [53]. However, in terms of cyberbullying, the results are inconclusive. Some studies have highlighted that girls are more involved in cyberbullying [2,12], while others have reported that the prevalence—both as cyberattackers and cybervictims—is greater in boys [51,54], although some authors observed no differences [55].

In the case of family communication, most researchers agree that girls have greater communication problems with their mothers than boys [15,21,56]. In relation to psychological distress, most studies have concluded that girls present higher indices of psychological distress during adolescence, mainly due to the influence of other variables, such as sensitivity with respect to the interpretation and expression of emotions and empathy and also with generally higher prevalence in girls than in boys [33,57,58,59,60]. Regarding attitude towards institutional authority, previous authors have observed that adolescent boys have a positive attitude towards the transgression of social norms, unlike girls, who show higher levels of positive attitude towards institutional authority [35].

### The Present Study

**Figure 1:**
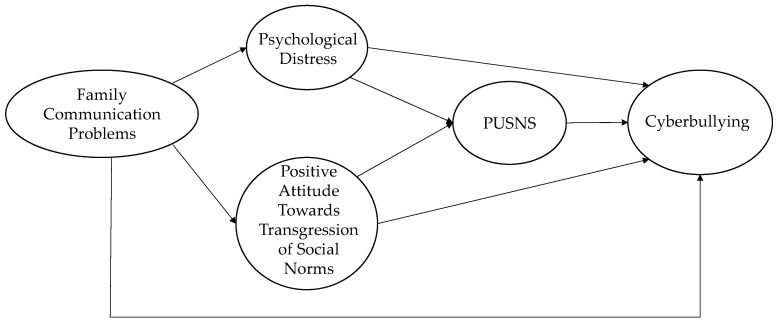
Theoretical model proposed.

Taking into account these precedents, the general aim of this study was to analyze the influence of family communication in cyberbullying, through its relationships with psychological distress, attitude towards the transgression of social norms and PUSNS. The following hypotheses were proposed: (1) family communication problems are directly related to cyberbullying; (2) family communication problems are indirectly related to cyberbullying through psychological distress and attitude towards the transgression of social norms; (3) psychological distress and attitude towards transgression are related with cyberbullying through PUSNS; and (4) gender differences exist in these relationships (see Figure 1).

## 2. Method

### 2.1. Participants

A stratified proportional sampling of urban and rural educational centers (universe of 984 centers) was conducted in the State of Nuevo León (Mexico) (confidence level 90%, alpha 0.05). A total of 8053 adolescents (51.5% boys and 48.5% girls) from 118 centers (62 urban and 56 rural) participated, of which 62.1% studied in urban schools and 37.9% in suburban schools. The ages ranged from 12–13 years (53.7%) to 14–16 years (46.3%). Data lost by scale or subscale, provided they did not exceed 15%, were treated using the multiple linear regression imputation model [61,62]. Univariate atypical data were detected by exploration of standardized scores [63].

### 2.2. Instruments

*Family Communication Problems Scale*. The offensive communication subscale of the Parent–Adolescent Communication Scale (PACS) [64] was used. This Likert-type subscale (e.g., “He/she tries to offend me when he/she gets angry with me”) consists of 10 items: communication with the mother and communication with the father, each with five response options (1 = never, 5 = always). Cronbach’s alpha was 0.73 (father) and 0.73 (mother). By means of a confirmatory factorial analysis (CFA) of the method of maximum likelihood, the two-dimensional structure of the scale was verified, showing a good or adequate adjustment to the data (mother (SB χ^2^ = 2594.5748, gl = 128, *p* < 0.001, CFI = 0.953, RMSEA = 0.049 (0.047, 0.051)); father (SB χ^2^ = 2885.3985, gl = 120, *p* < 0.001, CFI = 0.947, RMSEA = 0.053 (0.052, 0.055)).

*Cyberbullying Scale*. CYB–AGRESS [9] this consists of 10 items with five Likert-type response options, from 1 (never) to 5 (very often). The items measure the adolescent’s experience as an aggressor in the last 12 months, through the internet (e.g., “I have insulted or made fun of someone”) and cell phone (e.g., “I have made calls but I have not answered or I have asked someone to connect and I have not responded”). Cronbach’s alpha was 0.85. For the purpose of testing the factorial structure of the scale, a CFA was performed in this study, which showed a good fit to the data. (SB χ^2^ = 173.4424, gl = 32, *p* < 0.001, CFI = 0.950, RMSEA = 0.023 (0.020, 0.027)).

*Psychological Distress Scale*. K10 [24]. The scale consists of 10 Likert-type items with five response options (1 = never, 5 = always) that assess depressive and anxiety symptoms (e.g., “How often did you feel restless or fidgety”). The validation in Mexican population was found a Cronbach coefficient alpha of 0.90 and a unifactorial structure. [65]. For this research, a single scale dimension was also obtained through CFA using the maximum likelihood estimation method, obtaining a good fit to the data (SBχ^2^ = 504.7299, gl = 29, *p* < 0.001, CFI = 0.981, RMSEA = 0.045 (0.042, 0.049)).

*Positive Attitude towards the Transgression of Social Norms Scale*. The positive attitude towards transgression of social norms subscale of the Attitudes Towards Parents and Institutional Authority During Adolescence Scale (AAI-A) [66] was used. This Likert-type subscale (e.g., “If you do not like a school rule, it’s best not to follow it”) consists of 4 items, with four response options (1 = totally disagree, 4 = totally agree). Cronbach’s alpha was 0.74 and 0.74, respectively. For the purpose of verifying the factorial structure of the scale, CFA was performed in this study, which showed a good fit to the data (SB χ^2^ = 317.9209, gl = 23, *p* < 0.001, CFI = 0.976, RMSEA = 0.040 (0.036, 0.044)).

*Problematic Use of Social Networking Sites Scale* (PUSNS) [46]. This scale consists of 13 items, with answers ranging from 1 (never) to 4 (always). It measures the problematic use of social networking sites (e.g., “I need to be connected to my social networks continuously”). Cronbach’s alpha for this scale was 0.77. The CFA showed a good fit with the data (SB χ^2^ = 42.1864, gl = 2, *p* < 0.001, CFI = 0.990, RMSEA = 0.050 (0.038, 0.064)).

### 2.3. Procedure

The planning and research were carried out by researchers from the Autonomous University of Nuevo León in collaboration with the Pablo de Olavide University (Spain). After obtaining the permits and the active consent of the students, parents, and teachers, the instruments were administered in the selected centers under the supervision of the research staff. Participation was voluntary and anonymous, with a rejection rate of 0.21%. The study fulfilled the ethical values required in research with human beings, respecting the fundamental principles included in the Helsinki Declaration [67], in its updates, and in the regulations in force (informed consent and right to information, protection of personal data and guarantees of confidentiality, non-discrimination, free of charge, and the possibility of abandoning the study in any of its phases).

### 2.4. Data Analysis

Firstly, the Pearson correlations were calculated between all the variables studied and the t-test was performed. Then, an EQS 6.0 structural equations model [48] was calculated to analyze the relationship between the latent factors. Robust estimators were used to calculate the goodness-of-fit of the model and the statistical significance of the coefficients. IFC, IFI, and NNFI indices with values equal to or greater than 0.95 were considered acceptable, and for the RMSEA index values equal to or less than 0.08 [49]. Once the model had been calculated with the general sample of adolescents, a multi-group analysis was carried out to examine the differences in the relationships obtained according to the sex of the adolescents. Two groups were established according to the sex of the subjects: boys (N = 4177) and girls (N = 3938). Two models were calculated for the group of boys and girls, respectively. In the calculation of the first model (restricted model), all relationships between the variables had to be equivalent. By contrast, the second model (non-restricted model) was calculated without restrictions in the estimated parameters; hence, the relationships between the variables could differ in the different groups. Subsequently, the chi-square coefficient of the restricted and unrestricted models was compared; if this coefficient was significantly greater in the restricted model, invariance between the groups could be assumed.

## 3. Results

Table 1 shows the means, standard deviations, correlations between the variables studied, and t-test values as a function of gender. The correlation analysis showed significant relationships between the variables studied, so they were incorporated in subsequent analyses. The results obtained for the t-test revealed that the boys obtained higher scores in attitude towards the transgression of social norms and cyberbullying, whereas the girls showed greater problems of communication with their fathers and mothers, psychological distress, and PUSNS than the boys.

A structural equations model was then calculated with the EQS 6.0 program [68]. Table 2 shows the latent variables included in the model, their respective indicators, the standard error, and the associated probability for each indicator in the corresponding latent variable.

**Figure 2:**
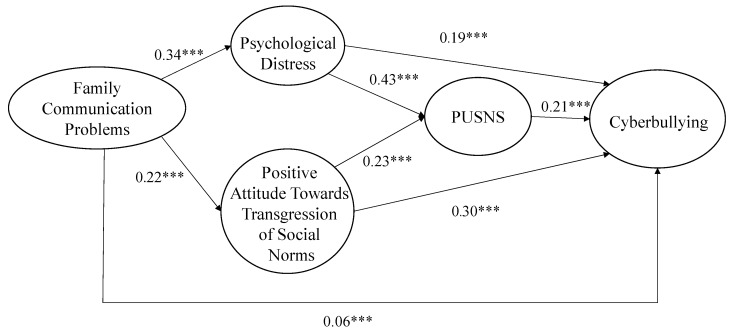
Final structural model with relation coefficients and statistical significance; *** *p* < 0.001.

With respect to the calculated equation model, the maximum likelihood method was used, with robust estimators (Mardia coefficient = 184.99; Normalized estimator = 326.57). The model showed a good fit with the data (S-B χ^2^ = 595.11, gl = 111, *p* < 0.001, NFI = 0.98, NNFI = 0.98; CFI = 0.98, RMSEA = 0.023 (0.021, 0.025) and explained 26.8% of the variance of cyberbullying. As shown in Figure 2, the results indicated that family communication problems were directly and positively related to cyberbullying (β = 0.06, *p* < 0.001), psychological distress (β = 0.34, *p* < 0.001), and a positive attitude towards the transgression of social norms (β = 0.22, *p* < 0.001). Psychological distress was directly and positively related to PUSNS (β = 0.43, *p* < 0.001) and cyberbullying (β = 0.19, *p* < 0.001). Likewise, a positive attitude towards the transgression of social norms was directly and positively related to PUSNS (β = 0.23, *p* < 0.001) and cyberbullying (β = 0.30, *p* < 0.001). Finally, PUSNS was directly related to cyberbullying (β = 0.21, *p* < 0.001).

In terms of the indirect effects (see Table 3), it was observed that family communication problems were related to cyberbullying through psychological distress and PUSNS (β =.030, IC (0.015–0.046), *p* < 0.001) and through psychological distress (β = 0.063, IC (0.047–0.079), *p* < 0.001). Likewise, family communication problems were related to cyberbullying through a positive attitude towards the transgression of social norms and PUSNS (β = 0.010, IC (0.0049–0.015), *p* < 0.001) and a positive attitude towards the transgression of social norms (β = 0.066, IC (0.0055–0.077), *p* < 0.001).

### Analysis of the Moderating Effect of Sex

After calculating the model, multi-group analyses were conducted to examine the effect of the gender of the adolescents on the relationships in the calculated model. The results showed significant differences in the model between boys (N = 4150) and girls (N = 3903) (Δχ^2^ (20, N = 8053) = 294.86; *p* < 0.001). After examining the restricted model, 7 restrictions were released. Four restrictions alluded to differences in the errors of the variables making up the model; hence, no substantial change was observed with respect to the relationships obtained in the general model. The fifth restriction indicated that the relationship between family communication problems and psychological distress was greater in girls (β = 0.43; *p* < 0.001) than in boys (β = 0.24; *p* < 0.001). The sixth restriction referred to the relationship between psychological distress and cyberbullying, which was greater in boys (β = 0.26; *p* < 0.001) than in girls (β = 0.18; *p* < 0.001). The seventh restriction indicated that the relationship between psychological distress and problematic use of social networking sites was greater in girls (β = 0.44; *p* < 0.001) than in boys (β = 0.32; *p* < 0.001). Once the restrictions had been released, both models showed that they were equivalent for boys and girls (Δχ^2^ (13, N = 8053) = 21.54; *p* > 0.05).

## 4. Discussion

The present study aimed to analyze the relationship between family communication problems and cyberbullying through psychosocial adjustment—psychological stress, attitude towards institutional authority and problematic use of social networking sites—in adolescents. The results showed a direct and significant relationship between the quality of parent–child communication and cyberbullying, thus confirming the first hypothesis. These results coincide with those published by other authors [4,22,69]. Additionally, family communication problems were indirectly related to cyberbullying through their relationships with psychological distress. These results are in line with those reported in other studies that have underlined that when family communication is based on insults, humiliating language, and aggression, children present symptoms related to psychological distress [26,70,71]. These negative emotions resulting from a negative family atmosphere, in which communication is cold and not very empathetic, are associated with greater engagement in cyberbullying behavior, as shown by the results obtained in this study. Although previous studies have reported that psychological distress is associated with higher levels of cyberbullying [72,73,74], the results obtained here introduce an interesting idea related to the role of family in the relationship between these two dimensions. These results could be attributed to the effects of poor family functioning—related to communication problems—on adolescents’ social skills [75] which, in turn leads to greater psychological distress [76], which is associated with higher levels of engagement in cyberbullying behaviors [73], as explained in the social skills deficit vulnerability model. Also, it could be argued that this style of interaction between parents and children, based on offensive communication as learned in the family environment, is also transferred to other scenarios, such as the field of peers. However, it would be worthwhile studying these relationships in greater depth in future research.

Additionally, the obtained findings show that communication problems between parents and children also seem to contribute to the development of a positive attitude towards the transgression of social norms, which is associated with greater involvement in delinquent behaviors, such as cyberbullying, thus confirming the second hypothesis. These results are consistent with those obtained in other studies highlighting the relationship between family communication problems and attitude towards institutional authority [16,77]. Indeed, offensive communication—a symptom of maladaptive family functioning—is associated with behavioral problems in children, as described in previous research [56,78]. Among these problems, one of the most serious relates to adolescents’ attitude towards figures of authority and the transgression of social norms which, in this case, is imposed in the family environment. Thus, adolescents who feel that they cannot share their emotions and thoughts with their parents in a positive and affectionate way are at greater risk of displaying a defiant and transgressive attitude towards them, in response to such poor family functioning [17,35,36].

Moreover, these results show that psychological distress and a positive attitude towards the transgression of social norms are related to cyberbullying through PUSNS, thus confirming the third hypothesis. In terms of the role played by PUSNS in the association between psychological distress and cyberbullying, the results obtained in this study were consistent with those published in previous research reporting a direct relationship between online behavioral problems, such as PUSNS, and cyberbullying [51,79,80]. In fact, recent research has underlined that PUSNS is a risk factor for cyberbullying and cybervictimization [46,81,82,83]. More specifically, it has been observed that spending an excess amount of time on online platforms, where social relationships are or may be problematic, is another factor that can increase the likelihood of cyberbullying or cybervictimization behavior [80,84].

A possible explanation for these results is that adolescents, whenever they feel psychological distress and at the same time perceive little support and understanding from their parents for prolonged periods of time, are also at greater risk of presenting behavioral problems related to social isolation and the compulsive search for new socialization environments in which interpersonal relationships are more satisfactory [85,86]. In this context, internet-based socialization platforms or social networking sites, which can be easily accessed by adolescents from their personal technological devices, e.g., smart phones, provide a context of social and emotional support in the virtual environment. In this scenario, it is more likely for adolescents to develop PUSNS when they display deficits in other socialization scenarios, such as family. In addition, a recent study by [87] highlighted that adolescents with PUSNS reported symptoms of psychological distress, such as trouble sleeping, anxiety, and depression.

One interesting finding in this study refers to the relationship between the attitude towards the transgression of social norms and cyberbullying through PUSNS. A recent study found that adolescents with more favorable attitudes towards the transgression of social norms reported a higher levels of PUSNS [33,83]. Numerous studies have highlighted the relationship between the attitude of adolescents towards institutional authority and problematic externalizing behaviors (such as criminal behavior) and addictions (such as alcohol and other substance abuse) [34,88,89,90,91]. This study makes an important contribution to previous studies, namely, the observation that the transgressive attitude towards social norms displayed by adolescents as a consequence of problematic family communication is a risk factor for PUSNS. In other words, PUSNS acts as an enabling factor for cyberbullying because adolescents perceive the internet as an escape route for their problems and a tool to challenge the rules imposed by their parents, which makes them more vulnerable and less capable of self-control. Given that the prevalence of PUSNS is increasing at a disturbing rate, more in-depth research should be conducted in this area in the future.

Finally, the results of the multi-group analysis revealed gender differences in some of these relationships, thus confirming the fourth hypothesis. Firstly, it was observed that the relationship between family communication problems and psychological distress was greater in girls than in boys. This result is consistent with the findings presented in previous studies highlighting that girls suffer greater problems of psychological distress, especially when they report problems of communication with their parents [12,92,93]. The aforementioned finding could be attributed to the fact that girls generally display greater sensitivity to family functioning problems, most likely due to implicit socialization practices [94].

Although previous studies have shown that boys engage in cyberbullying behaviors more frequently than girls [9,95,96,97], the results obtained in the present study show that although boys actually claim to partake more in cyberbullying behaviors than girls, the importance of psychological distress in cyberbullying, as a consequence of problematic family communication, is greater in the latter. We believe this result should be explored in greater depth in future research in so far as it suggests that in the case of girls, involvement in cyberbullying is associated with greater difficulty in dealing with communication problems in the family environment. Additionally, psychological distress is more strongly related to PUSNS in girls than in boys, suggesting that girls, in situations of distress, are more likely to use social networking sites as a way to obtain social support from their peers and establish new friendly relationships that minimize the adverse effects of psychological distress and difficulties in the family. This finding can be considered highly significant and these relationships should be analyzed in greater depth in future research.

The present study had the following limitations. Its transversal nature prevented the establishment of causal relationships, so it would be interesting to incorporate the time dimension in future studies. Also, it would be interesting in future research to carry out a multilevel analysis to know the effect of the centers on the relations analyzed. Another limitation is that indirect effects are small due to how the calculation is made; nevertheless, they are significant, and taking into account the size of the sample, they provide relevant results for the study as a whole. Self-reporting measures were used to obtain data. When the researchers instructed the adolescents participating in the study to complete the cyberbullying questionnaire, they informed them that they should respond thinking of their peers (classmates and/or friends of their peer group). In future studies, it would be interesting to use other types of measurements that take into account parents’ perceptions, in order to obtain more in-depth knowledge of the relationships between the variables.

## 5. Conclusions

This study highlights the importance of family in cyberbullying behaviors, in so far as it has a direct and indirect effect on cyberbullying. Communication problems seem to be an important source of psychological distress, and are expressed in the form of higher levels of PUSNS and cyberbullying, especially in girls. In addition, these family problems seem to explain the attitude towards the transgression of social norms, increasing the likelihood of adolescents engaging in both PUSNS and cyberbullying. We consider the practical implications derived from our work, since the obtained results provide ideas for the promotion of programs aimed at promoting the responsible use of new technologies since, in many cases, adolescents are not aware of the psychological and social consequences of cyberbullying. It would also be important to work on intervention in families in order to improve relations between parents and children and promote open and positive communication in the family. In summary, we consider it of interest to work on resources that enable empowerment in adolescents, so that they do not become involved in risky behavior, such as cyberbullying.

## Figures and Tables

**Table 1:** Means, Pearson correlations, standard deviations, and Student’s t.

Variables	1	2	3	4	5	6
PCM	1					
PCF	0.69 **	1				
PD	0.34 **	0.25 **	1			
PATTSN	0.18 **	0.14 **	0.13 **	1		
PUSNS	0.25 **	0.18 **	0.38 **	0.22 **	1	
CB	0.23 **	0.16 **	0.29 **	0.32 **	0.31 **	1
M/SD boys	1.9/0.8	1.9/0.8	1.8/0.7	1.6/0.7	1.7/0.6	1.3/0.4
M/SD girls	2.1/0.8	2.0/0.8	2.3/0.9	1.5/0.6	1.9/0.7	1.2/0.3
*t*	−8.34 ***	−5.03 ***	−23.90 ***	9.31 ***	−16.63 ***	5.71 ***

Notes: ** *p* < 0.01; *** *p* > 0.001; PCM: problematic communication mother; PCF: problematic communication father; PD: psychological distress; PATTSN: positive attitude towards transgression of social norms; PUSNS: problematic use of social networking sites; CB: cyberbullying.

**Table 2:** Factorial saturations, standard error, and associated probability.

Variables	Factor Loading General Model
Family communication problems	
Problematic communication mother	1 ^a^
Problematic communication father	1.56 *** (0.05)
Psychological distress	0.768 *** (0.04)
Positive attitude towards transgression of social norms	0.071 *** (0.01)

Note: *** *p* < 0.001. Robust statistic. Standard errors in brackets. ^a^ Fixed in 1.00 during simulation.

**Table 3:** Indirect effect, direct, and total effects of the total model.

	β	Standard Error(s)	*p*	C.I. 95%	
				LCL	UCL
**Indirect effects**					
FCP→PD→PUSNS→Cyberbullying	0.030	0.0081	<0.001	0.015	0.046
FCP→PD→Cyberbullying	0.063	0.0081	<0.001	0.047	0.079
FCP→PATTSN→PUSNS→Cyberbullying	0.010	0.0027	<0.001	0.005	0.015
FCP→PATTSN→Cyberbullying	0.066	0.0058	<0.001	0.055	0.077
**Direct effects**					
FCP→Cyberbullying	0.059	0.018	<0.001	0.024	0.094
**Total effects**					
FCP→Cyberbullying	0.229	0.025	<0.001	0.18	0.278

Notes: Total effect is the sum of direct effect of family communication problems to cyberbullying and its indirect or mediating effects. FCP = family communication problems, PD = psychological distress, PUSNS = problematic use of social networking sites. PATTSN = positive attitude towards transgression of social norms; *p* < 0.001.
